# Review of "*Molecular Mechanisms of Parasite Invasion" *by Barbara A. Burleigh and Dominique Soldati-Favre

**DOI:** 10.1186/1756-3305-2-24

**Published:** 2009-05-05

**Authors:** Kevin M Tyler

**Affiliations:** 1 BioMedical Research Centre, School of Medicine, Health Policy and Practice, University of East Anglia, Norwich, NR4 7TJ, UK

## Abstract

Book review of "*Molecular Mechanisms of Parasite Invasion" *by Barbara A. Burleigh and Dominique Soldati-Favre

## Review

Medical microbiologists inhabit a post-genomic environment of platform technologies engendering large datasets of host and pathogen components. An environment in which there is extensive knowledge of the DNA, RNA and protein of key parasites and of human cells. This offers the opportunity for functional studies with unprecedented definition and has led to the blossoming of cellular studies investigating how pathogens interact with host cells and in particular how relatively large and complex protozoa can actually enter, survive and proliferate within the cells of human hosts.

Several lineages of parasite have adopted intracellular strategies for survival, presumably driven by similar selective pressures for immune evasion and sustenance. To adopt an intracellular life-style, parasites must find ways to enter host cells, overcome their innate cellular defences and divert their nutritional resources. Readers of this book will discover that while many of the exact mechanisms by which this is achieved vary from parasite to parasite and from parasitized cell to parasitized cell; there are instances of mechanistic convergence and common threads to the mechanisms adopted by different parasites.

As someone working in this field I found this book enormously interesting and useful, drawing together as it does a number of different systems to facilitate comparisons by the reader. Active researchers and PhD students in this and affiliated fields will no doubt find this an essential resource for several years to come. Essentially, the book is an edited compilation of highly technical reviews from many of those who founded this area of research. In terms of readability, the book is light-weight and portable, but this reflects the fact that the text is extremely dense and the font size small, making reading a little difficult. It is though well illustrated and incorporates appropriate colour and black and white images which improves the overall readability and facilitates understanding of the quite complex subjects covered.

The book is broken down by sections and chapters; with sections on experimental approaches, receptor interactions, signalling pathways, cytoskeletal dynamics, proteases, parasitophorous vacuole and penetrating biological barriers. It kicks off with a chapter devoted to contemporary methods for looking at apicomplexan cell invasion which is generally useful. Subsequent sections contain multiple chapters, each organism-specific, facilitating comparisons. This structure enables the reader to do the hard work of finding common themes between systems rather than being spoon-fed them; presumably because for the diligent and interested reader the best understanding is earned rather than given.

## Competing interests

The author declares that they have no competing interests.

